# 18q21.1q21.32 Deletion in a Patient With Juvenile Cerebral Infarction

**DOI:** 10.7759/cureus.42534

**Published:** 2023-07-27

**Authors:** Koji Obara, Takumi Inomata

**Affiliations:** 1 Neurology, National Hospital Organization Akita National Hospital, Yurihonjo, JPN; 2 Neurology, Yasumi Hospital, Morioka, JPN

**Keywords:** tcf4, striatocapsular infarction, pitt–hopkins syndrome, 18q deletion, acgh

## Abstract

The chromosome 18q deletion syndrome is a well-recognized chromosomal aberration characterized by intellectual disability, facial dysmorphism, short stature, microcephaly, cardiac anomalies, such as atrial and ventricular septal defect, and hypotonia; however, the phenotype is highly variable depending on the combination of genes within the chromosomal aberration regions. Thus far, no association was found between 18q deletion and cerebral infarction. Herein, we report a case of 18q deletion syndrome that caused juvenile cerebral infarction. A 32-year-old woman with an intellectual disability and facial dysmorphism presented with sudden-onset left-sided weakness. Brain magnetic resonance imaging revealed a striatocapsular infarction. Abnormalities in thrombotic profiles and embolic sources could not be identified. Microarray-based comparative genomic hybridization analysis detected a microdeletion in chromosome 18 encompassing the cytoregion 18q21.1q21.32. The deletion region contains the *TCF4* and *SMAD4* genes, whose haploinsufficiency causes the causative genes of Pitt-Hopkins syndrome (PTHS) and juvenile polyposis/hereditary hemorrhagic telangiectasia (JPHT or JPHHT), respectively. The patient’s facial features were characteristic of PTHS, including a broad, beaked nasal bridge and a wide mouth with a bow-shaped upper lip. On the contrary, the patient did not show breathing abnormalities, which is one of the hallmarks of PTHS. We could not elucidate the relationship between cerebral infarction and genes included in the deleted region of 18q. However, if patients with chromosomal aberrations have cerebral infarctions, investigating the genes included within the chromosomal aberration regions may increase our knowledge of the genes involved in juvenile cerebral infarction.

## Introduction

To date, cases of juvenile cerebral infarction have been reported in patients with various numerical and structural chromosomal aberrations [1-5]. Some of these cases have thrombus-related genes within chromosomal aberration regions, such as the *VWF* gene encoding von Willebrand factor and the *SERPINC1* gene encoding antithrombin [1, 2]; however, in others, the association between chromosomal aberrations and cerebral infarction remains unclear. The 18q deletion syndrome (OMIM 601808) is a well-recognized chromosomal aberration, with an estimated prevalence of approximately 1 in 40,000 live births [6]. The phenotype is highly variable, including an intellectual disability, facial dysmorphism, short stature, microcephaly, cardiac anomaly, and hypotonia [6-8]. Herein, we report the case of a female patient with an 18q21.1q21.32 deletion and cerebral infarction developed at the age of 32.

## Case presentation

A 32-year-old woman with intellectual disability and facial dysmorphism visited our institution with left-sided weakness that had developed the day before. She was born to non-consanguineous parents. Her mother’s pregnancy and delivery were normal. Her birth weight was approximately 2,500 g. Birth length and head circumference were unknown. From birth onward, she had feeding problems, including poor suckling and delayed motor development. She could sit on the carpet without assistance and stand with help but could not walk. From an early age, she exhibited stereotypic flapping movement of hands. She had no language development and never uttered a word. In her late teens, she began to have epistaxis, which has recently increased in frequency to about once every two weeks. She did not have any risk factors for atherosclerotic disease, such as hypertension, hyperlipidemia, and diabetes.

**Figure 1:**
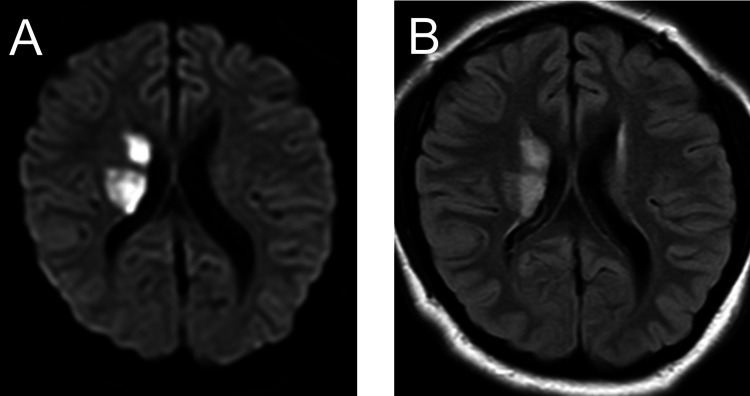
Brain magnetic resonance imaging (A) Diffusion-weighted imaging and (B) fluid attenuation inversion recovery imaging show a high-intensity lesion involving the right putamen, anterior limb of the internal capsule, and part of the head of the caudate nucleus.

Physical examination revealed a temperature of 36.7°C, pulse rate of 109 beats/min, blood pressure of 162/120 mmHg, height of 140 cm, weight of 36 kg, and head circumference of 47 cm. Heart sounds were clear, and rhythm was regular without audible murmurs. She had dysmorphic facial features, including a deep-set eye, strabismus, broad and beaked nasal bridge, wide mouth with bow-shaped upper lip and an everted lower lip, widely spaced teeth, and low-set ears. Moreover, she had scoliosis and over-riding toes. On neurological examination, she was alert but did not utter words or follow instructions, her eye gaze was normal, and she showed slight signs of left central-type facial palsy. Babinski sign was positive bilaterally, and she exhibited hypotonia in the left upper and lower extremities without voluntary movements. Brain magnetic resonance imaging revealed a lesion involving the right putamen, anterior limb of the internal capsule, and part of the head of the caudate nucleus with a high signal on fluid attenuation inversion recovery imaging and diffusion-weighted imaging consistent with an acute ischemic stroke (Figure 1).

MR angiography did not show any stenosis or occlusion in the main cerebral arteries. Thoracic echocardiogram showed no abnormal findings such as intracardiac thrombus and patent foramen ovale. Transesophageal echocardiography was not performed. The 72-h electrocardiogram gave a normal tracing. Contrast-enhanced thoracic computed tomography (CT) did not reveal any pulmonary arteriovenous malformations. Thrombotic profiles including homocysteine, antithrombin III, and protein C and S activities were within the normal range. Immunological tests including lupus anticoagulant, anticardiolipin antibodies, antinuclear antibodies, antiphospholipid antibodies, anti-SS-A/B antibodies, and myeloperoxidase/proteinase 3-antineutrophil cytoplasmic antibodies were negative. She received combination therapy involving argatroban, clopidogrel, and cilostazol. Her left hemiparesis gradually improved. No residual symptoms were observed 9 months after the stroke onset.

**Figure 2:**
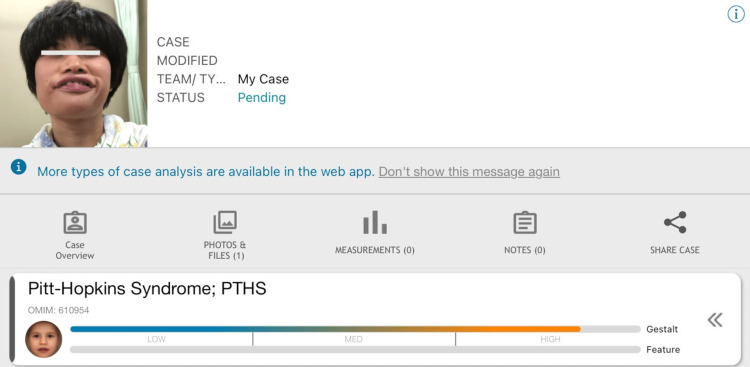
The analysis of the patient's face using Face2Gene The face of the patient with a deep-set eye, broad and beaked nasal bridge, and wide mouth with a bow-shaped upper lip and an everted lower lip. Face2Gene shows that the patient's face has a high gestalt score to Pitt–Hopkins syndrome.

The following analyses were ordered to determine the etiology of facial dysmorphisms with an intellectual disability and juvenile cerebral infarction. First, Face2Gene (FDNA Inc., Boston, USA) was used to analyze the patient’s face and obtained high gestalt in Pitt-Hopkins syndrome (PTHS) (Figure 2).

**Figure 3:**
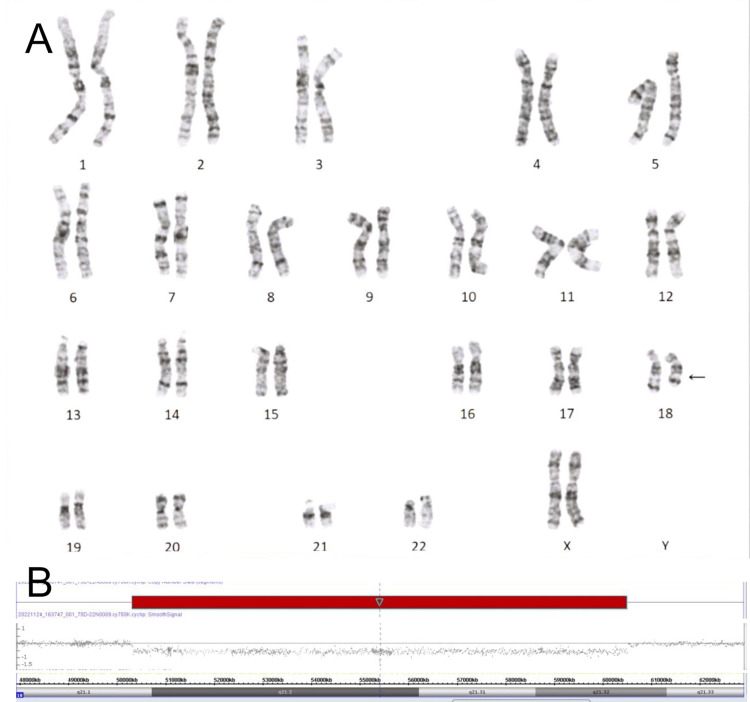
Karyotype and array analyses of the patient (A) The conventional karyotyping (400–550 band level) indicated 46,XX,del(18)(q21.1q21.3). (B) Microarray-based comparative genomic hybridization analysis indicated a 10.1 Mb (50,300,380- 60,488,724) deletion in 18q21.1q21.32.

Then conventional karyotyping was performed, which revealed an abnormal karyotype 46,XX,del(18)(q21.1q21.3) (Figure 3A). Finally, to identify the genes included in this deleted region of 18q, a microarray-based comparative genomic hybridization (aCGH) was applied in the following methods. Genomic DNA from the peripheral venous blood sample was obtained using standard techniques and hybridized to a Cytoscan 750K Suite microarray (Affymetrix, Inc., Santa Clara, USA), in accordance with the manufacturer’s instructions. After hybridization, the microarray was washed, stained, and scanned with a GeneChip Scanner 3000Dx v. 2 (Affymetrix, Inc.). Allele and intensity ratio data of the fluorescent signals were generated. Microarray data were visualized and analyzed using Chromosome Analysis Suite version 4.3.0.71 (Affymetrix, Inc.) to identify copy-number variations and regions with the absence of heterozygosity. As a result, the aCGH analysis has detected a deletion of 10.1 Mb in chromosome 18 encompassing the cytoregion 18q21.1q21.32: Arr[GRCh38] 18q21.1q21.32(50300380_60488724)×1 (Figure 3B).

This region contains 58 RefSeg genes, of which 36 are included in the Online Mendelian Inheritance in Man (OMIM) database. Among these genes, *TCF4* and *SMAD4 *cause different haploinsufficiency syndromes.

A written informed consent was obtained from the parent of the patient for the use of blood samples in the genetic analysis, a photograph of the patient's face that was uploaded to the Face2Gene application for analysis, and publication of details of the medical case and any accompanying images including photographs of the patient. The study was conducted in accordance with the principles of the Declaration of Helsinki.

## Discussion

Herein, we reported the case of a patient with intellectual disability and facial dysmorphisms, who suffered juvenile cerebral infarction and was later identified to have 18q21.1q21.32 deletion. In our search of English articles in PubMed, no articles have described an association between the 18q deletion syndrome and cerebral infarction. The 18q deletion in our patient encompassed the whole *TCF4* gene and *SMAD4* at 18q21.2. *TCF4* haploinsufficiency causes PTHS, which is characterized by severe intellectual disability, facial dysmorphisms, and breathing abnormality [9]. Indeed, in our patient, her facial dysmorphisms showed a high gestalt score to PTHS by Face2Gene. On the contrary, she did not show breathing abnormalities. In a previous report, breathing abnormality, one of the hallmarks of PTHS, was found in only approximately half of patients with 18q deletion and *TCF4* hemizygosity [10]. On the contrary, *SMAD4* haploinsufficiency induces juvenile polyposis/hereditary hemorrhagic telangiectasia (JPHT or JPHHT) [11]. Our patient’s frequent epistaxis from childhood may be one of the symptoms of JPHT. Moreover, JPHT can cause visceral arteriovenous malformations (AVMs); however, our patient did not have any visceral AVMs, including pulmonary AVMs [11].

Our patient had an infarction over almost the entire territory of lenticulostriate arteries, the so-called striatocapsular infarction (SCI). The pathomechanisms of SCI include artery-artery embolism from the internal carotid artery and cardiogenic embolism and atherosclerotic disease in the middle cerebral artery at the origin of the lenticulostriate arteries [12]. To examine for cardiogenic embolism, our patient had no arrhythmias, such as atrial fibrillation or cardiac disease, evident on electrocardiogram and transthoracic echocardiography. However, she did not undergo transesophageal echocardiography; thus, we could not exclude the presence of patent foramen ovale or atrial thrombus. *SMAD4* haploinsufficiency, JPHT, can produce a paradoxical cerebral embolism derived from a pulmonary AVM; however, our patient did not have a pulmonary AVM detected on contrast-enhanced CT. We could not find known genes associated with thrombogenicity and ischemic vulnerability within the deleted region.

## Conclusions

We reported the case of a young adult patient with 18q21.1q21.32 deletion who had a SCI. Her facial dysmorphisms were similar to those of PTHS. In this case, we could not elucidate the relationship between genes included in the deleted 18q region and cerebral infarction. However, if patients with chromosomal aberrations have cerebral infarctions, investigating the genes included within the chromosomal aberration region may increase our knowledge of the genes involved in juvenile cerebral infarction.
